# Case of the Bite of a Mad Dog and Its Treatment

**Published:** 1846-06

**Authors:** Daniel Stahl

**Affiliations:** Quincy, Illinois


ARTICLE IV.

*Case of the Bite of a Mad Dog, and its treatment.* By DANIEL STAHL, M. D., of Quincy, Illinois.

There is, perhaps, no disease in the whole register of nosology, that is more dreadful in its manifestations, and more certain in its destruction, than hydrophobia; and none, I may add, that is more obscure to the pathologist, and less under the control of medical agents, than this awful disease. Much, very much, has been written on hydrophobia, many remedies have been lauded as infalliable in its cure, and many, if not all of them have disappointed us in the hour of need,—none have as yet stood the test of time, and I much doubt whether as yet we possess, out of the many hundreds of "curative plans" for this scourge of the animal organization, a single one on which we can rely with confidence. Accident, or organic chemistry, are the two sources to which I look for aid in this disease, and as long as we cannot *cure* it, we must more earnestly endeavor to *prevent* it. Can we prevent it when the hydrophobic poison is once introduced into the animal system? In answer to this question I take the liberty to relate the following case, and leave it to the reader's own judgment to answer it, remarking merely, that it is now nearly 15 months since, the patient was bitten by a dog, which, from the following reasons, I considered rabid.

1. The appearance of the dog, as described by the patient and his father, who is a very intelligent gentleman.

2. The irresistable desire of the dog to bite every thing and every body.

3. His constant restlessness and running until he was killed; and finally and principally

4. *The manifestation of undoubted symptoms of Hydrophobia in at least* 12 *animals* (dogs, hogs, and horned cattle,) *that were bitten by this dog on the same or on the subsequent day of that on which my patient was bitten by him.*

I will not impose upon the patience of my readers a long and minutely detailed history of the case, but will merely relate the main features as put down during the time of treatment in my case book. As an apology, (if apology is necessary) for this brevity, I will merely say, that I feel some embarrassment in writing in a foreign language; which the English language is to me.

Harvey Barr, a stout, healthy lad, of 14 years of age, was bitten on the morning (about 8 o'clock) of the 10th of February, 1845, by a dog supposed to be rabid. In the afternoon he came to my office. He had five wounds inflicted by the teeth of the dog on both sides of the middle and ring fingers. Some of these wounds consisted of but an abrasion of the skin, others penetrated through the skin. I washed the wounds with spirit. cornu cervi, and gave him a dose of Epsom salts, and ordered the wounds to be cauterized with a red-hot iron. This latter not having been done, I went to the place of his residence (6 miles from the city) and performed cauterization with a red-hot knitting-needle, about 10 o'clock, P. M. I then put ungent. cantharid. on the wounds, and ordered a pill of gr. ½ ext. belladonna, and gr. j. of ferr. carbon., to be taken thrice a day, until the pupil should become very much enlarged, when but one pill, daily, was to be taken.

*February* 26. The hog of Hiram Barr, supposed to have been bitten by the same dog that bit Harvey, became a few days ago hydrophobic, and died under the usual symptoms of that disease. This, of course, alarmed the father of Harvey, and he requested that I might consult some other physicians. Drs. Taylor and Bartlett, two well educated and intelligent gentlemen, were accordingly called in. The belladonna had manifested its wonted constitutional symptoms, such as itching, dryness of the throat, enlarged pupil, &c. The wounds are suppurating, and the hand is swollen and painful, but the pain does not extend beyond the hand. Patient is perfectly tranquil and happy, not appearing to be troubled with any apprehension of danger; he eats and sleeps well.

It was the opinion of Drs. Taylor and Bartlett to continue the belladonna, apply ungent. mercuri. instead of the ungent. cantharid., and use Epsom salts as a cathartic; all of which was, by me, ordered to be done.

*March* 2. The wounds did not suppurate well. Having just read the account of a similar case communicated by Dr. Hildreth, of Ohio,* in which he had used externally, hydrarg. precipitatum rubr. cum. cupr. sulph. I discontinued the ungent. mercuriale, and used Dr. Hildreth's prescription.

*March* 5. Heard of several dogs and hogs having been bitten by the same dog that bit Harvey. Dr. Bartlett and my-

* Medical Repository, Vol. VII.

self went to Joseph Turner's, in the neighborhood of Barr's, and became satisfied that his (Turner's) two dogs were rabid.

*March* 8. The wounds have dried up. Ordered emollient poultices and ungent cantharid., also three pills daily, each containing ½ gr. of ext. belladonna.

This treatment, i. e.: the application of the cantharides ointment, and the internal use of belladonna, with occasionally a dose of salts, I continued for three months till the 10th of May, when I healed up the suppurating wounds, and dismissed the patient. I have had, however, in the mean time a vigilant eye upon him, but could not detect the least symptom similar to any of those we see in hydrophobia.

April 30, 1846.

P. S. I am at this time attending a boy who was bitten by a rabid dog on the 10th of March, 1846. I treat him in the same manner as I did the subject of the above narrative, with the exception of the use of the cauterium actuale. In a year or two I shall communicate this case also, whatever may be the result of my treatment.

